# Ageing effects of social environments in ‘non-social’ insects

**DOI:** 10.1098/rstb.2022.0463

**Published:** 2024-10-28

**Authors:** Lauren M. Harrison, Emily R. Churchill, Megan Fairweather, Claire H. Smithson, Tracey Chapman, Amanda Bretman

**Affiliations:** ^1^ School of Biological Sciences, University of East Anglia, Norwich, Norfolk, NR4 7TJ, UK; ^2^ School of Biology, Faculty of Biological Sciences, University of Leeds, Leeds, LS2 9JT, UK

**Keywords:** lifespan, longevity, senescence, sociality, *Drosophila*

## Abstract

It is increasingly clear that social environments have profound impacts on the life histories of ‘non-social’ animals. However, it is not yet well known how species with varying degrees of sociality respond to different social contexts and whether such effects are sex-specific. To survey the extent to which social environments specifically affect lifespan and ageing in non-social species, we performed a systematic literature review, focusing on invertebrates but excluding eusocial insects. We found 80 studies in which lifespan or ageing parameters were measured in relation to changes in same-sex or opposite-sex exposure, group size or cues thereof. Most of the studies focused on manipulations of adults, often reporting sex differences in lifespan following exposure to the opposite sex. Some studies highlighted the impacts of developmental environments or social partner age on lifespan. Several studies explored potential underlying mechanisms, emphasizing that studies on insects could provide excellent opportunities to interrogate the basis of social effects on ageing. We discuss what these studies can tell us about the social environment as a stressor, or trade-offs in resources prompted by different social contexts. We suggest fruitful avenues for further research of social effects across a wider and more diverse range of taxa.

This article is part of the discussion meeting issue ‘Understanding age and society using natural populations’.

## 1. Introduction

That social interactions can have consequences for health and ageing is well-documented in humans and other animals with complex societies and social behaviours [1–9]. However, species typically considered ‘non-social’ may also have well-established and wide-ranging responses to variation in their social environment. This is because sociality is not a fixed set of social behaviours, but rather a spectrum covering simple to complex sets of social interactions [10–12]. At the simplest level, social interactions can refer to any interaction between conspecific individuals. At the other end of the sociality spectrum lie the eusocial insects, where kin selection has resulted in the evolution of complex social interactions occurring within a related social group. For example, there is division of labour among members of eusocial insect colonies, whereby closely related workers take on the bulk of resource gathering, offspring care and colony maintenance while the queen produces offspring [13,14]. Queens in some eusocial species are also remarkably long-lived, and it is suggested that it is their eusocial lifestyle that allows them to escape reproduction–lifespan trade-offs [15,16] (but see [17]). Thus, studies of eusocial insects have significantly shaped our knowledge of sociality, including its impacts upon ageing [13]. However, there are potential problems with an overreliance on social insects as models for ageing and lifespan. Queens living within protected, stable social environments face low extrinsic mortality risk, and hence slower rates of ageing [16]. The relatedness among individuals in eusocial groups may challenge their use as a general model to partition out the effects of social interactions *per se* separately from kin-selected effects. In this review, we address this by synthesizing across studies the effects of social interactions in lesser-studied non-social insect species to better understand the specific effects of social interactions on ageing and lifespan.

Studies in humans show a variety of impacts that social interactions may have on ageing patterns. Generally, negative social experiences, such as weak social connectedness [18], negatively correlate with health [19] and mortality risk [20]. However, this is not the case for all dimensions of health (e.g. [21]). For instance, the quality of social interactions (i.e. strength of the relationship) and an individual’s perception of their connectedness within a social network seem to be key indicators of cognitive function in older adults rather than just the frequency of social interactions [22]. Moreover, increasing evidence suggests that social interactions that impact upon ageing trajectories, lifespan [23] and ultimately fitness are not restricted to species that show strong social or familial bonds [24] (electronic supplementary material, table S2). Therefore, it is plausible that the social environment can have widespread effects on senescence and lifespan, and that these effects could be trait-, context- and even sex-specific [25]. To fully understand the generality of these patterns requires investigation across species that vary in their social complexity.

One such example is the laboratory stalwart *Drosophila melanogaster* fruit fly. *D. melanogaster* has been used extensively as a model in the field of ageing [26,27] and increasingly to understand social behaviour and the effects of the social environment. *Drosophila* species have been described as communal [10,28] because overlapping generations share the same space, but elsewhere they are described as solitary and lacking complex social behaviour [13]. However, a growing body of work suggests that *Drosophila* fruit flies are highly responsive to their social environments [28–31]. This includes impacts of the social environment upon fly health, such as immune responses [32] and cancer progression [33]. Chronic social isolation reduces sleep [34] (critical to cognitive function) and induces hunger, causing flies to overeat [35]. The above examples demonstrate that even in a species with no noted kin structure the social environment can have strong effects on life-history traits. This raises two important questions that we address here of (i) whether the degree of social interactions either within or across sexes has general effects on ageing and lifespan, and (ii) whether these effects are found more generally across other insect/invertebrate taxa.

### (a) Aims of this review

To test the hypothesis that the social environment significantly affects lifespan and ageing in non-social invertebrates, we performed a systematic review of the literature. Our objective was to assess the taxonomic breadth of investigations, the types of social manipulations being performed and how researchers quantify the effects of social environment on lifespan and rates of ageing. From this, we aimed to use the results to detect any emergent themes such as key drivers and trade-offs, and identify any potential shared mechanisms involved. Specifically, we addressed the following questions:

1. Do early life and/or adult social environments impact adult lifespan, potentially through trade-offs with other life-history traits?
2. Do interactions within or across the sexes have widespread and consistent effects on ageing and lifespan? This was based on the rationale that we might expect the sexes to respond differently to opposite sex exposure, owing to sex-specific costs of reproduction. However, it is unclear how same-sex or mixed-sex interactions might impact lifespan in both sexes and across species with varying mating systems and social tendencies.
3. What kinds of mechanisms are generally identified or tested in studies of the social environment, ageing and lifespan?

Although we included studies using manipulations of sex ratio, which inevitably causes differences in reproductive rates, hence lifespan, our aim was not to focus on costs of reproduction *per se*, discussed extensively elsewhere (e.g. [36]). Instead, we focused on how varying exposure to the same or the opposite sex (the sociosexual environment) can have sex-specific effects on lifespan and ageing. The interactions between sociality and ageing could be bidirectional. For example, individuals could change their social behaviour with age, as seen in humans (and non-human primates e.g. [37–41], could become more selective of social partners with age. However, we mainly focused here on the idea that the frequency or type of social interaction could alter lifespan and senescence.

## 2. Methodology

### (a) Literature search

To test the hypothesis that the social environment affects lifespan and ageing we conducted a systematic search of the literature across invertebrate taxa. We followed the systematic review guidelines suggested by Foo *et al*. [42]. We identified empirical studies that measured lifespan or ageing following a manipulation of social context. We conducted a keyword search using the *ISI Web of Science* online database on 6 July 2023 using the following search string: (("social environment*" OR "social interaction*" OR "indirect genetic effects" OR "social behaviour" OR "social behavior" OR "social competition" OR "social contact*" OR "same-sex competitors" OR "male–male" OR "female–female" OR "social effects" OR "social perception" OR "rival*" OR "sex ratio" OR "sexual perception" OR "solitary and group") AND ("stress resistance" OR lifespan OR "life span" OR "life-span" OR mortality OR age OR ageing OR aging OR longevity OR senescence) AND (invertebrate OR insect OR drosophila OR fly OR beetle OR cricket OR moth OR butterfly)). After filtering the search results to include research articles only, 1758 unique records remained for title/abstract screening.

### (b) Eligibility criteria

The review was restricted to studies of invertebrate taxa that include a measure of adult lifespan or ageing in response to the social environment. We included studies that manipulated the juvenile social environment if they then quantified lifespan or ageing in the subsequent adult life stage. We excluded studies during screening that (i) were reviews or theoretical papers, (ii) did not include a manipulation of the social environment, (iii) did not measure ageing or lifespan, (iv) investigated non-invertebrate taxa, or (v) focused on orders in which eusocial insects are found. We further excluded articles during full-text screening if they did not have suitable data on lifespan or longevity. Screening was carried out equally by all authors. After screening, we retained 80 eligible studies ([25,32,43–119]; electronic supplementary material, tables S12). The full search and inclusion/exclusion protocol is summarized in a PRISMA diagram (electronic supplementary material, figure S1). It should be noted that we only provide one reason for exclusion for each study, although studies could meet multiple exclusion criteria.

## 3. Results and discussion

### (a) Taxonomic diversity

**Figure 1.**
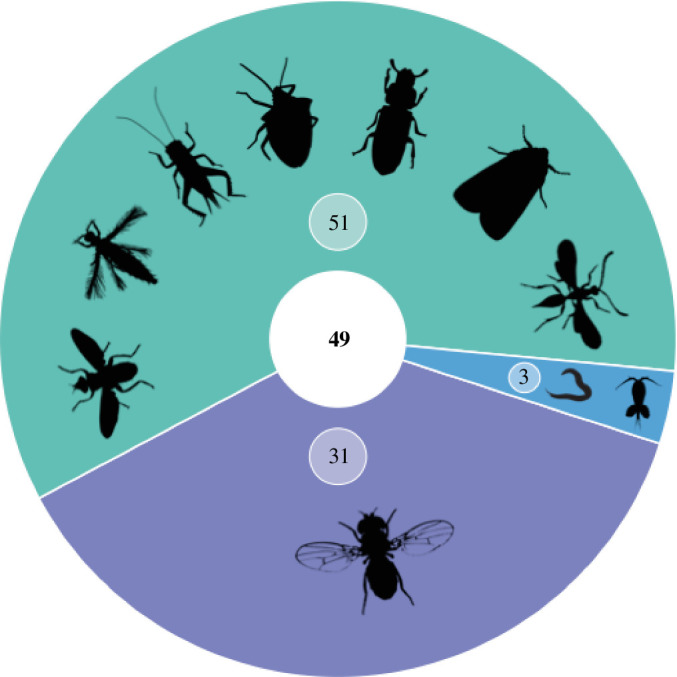
Taxonomic diversity observed across the 80 unique studies that tested how social environments influence lifespan or ageing in insects/invertebrates. The centre circle reflects the total number of unique species represented in the 80 studies. The smaller circles show the number of studies that use a *Drosophila* species (purple), another insect species (green) or an invertebrate species (blue). Silhouettes represent some of the invertebrate orders present in the different studies.

Our main aim was to formally identify the number of studies that have tested how the social environment influences lifespan or ageing in invertebrate species typically considered as ‘non-social’. Overall, we identified 80 such studies, covering 49 invertebrate species, that manipulated the social environment and measured its effect on lifespan or ageing (figure 1). *Drosophila* species (*D. melanogaster*, *D. prolongat*a, *D. serrata*, *D. simulans*, *D. grimshawi*, *D. subobscura* and *D. pseudoobscura*) dominated, with around 40% of studies utilising these model species (*n* = 31 studies). Other Dipteran species (e.g. *Ceratita capitata* fruit flies, *Telostylinus angusticollis* flies) and model Coleopteran species (e.g. *Tribolium castaneum* [116] and *Gnatocerus cornutu*s beetles [69,70]) were well represented (*n* = 11 and *n* = 10 studies, respectively) in the final set. Although our keyword search included other invertebrates, only 3 of the final 80 studies focused on non-insect invertebrates (the nematode worms: *Caenorhabditis elegans* [85], *C. remanei* [67] and the pelagic copepod: *Oithoca davisae* [92]).

### (b) Early life social environments and their impact on lifespan

Resource competition during development can determine how individuals allocate resources towards reproduction and lifespan. Exposure to favourable environmental conditions in early life, such as low competition for resources, is expected to have positive effects on fitness-related traits (i.e. the ‘silver-spoon’ effect [120]), while the opposite is true for unfavourable or stressful early life conditions [6]. As such, there can be strong developmental effects on adult phenotypic variation and social behaviour that can influence fitness and senescence (but see [121]).

**Figure 2.**
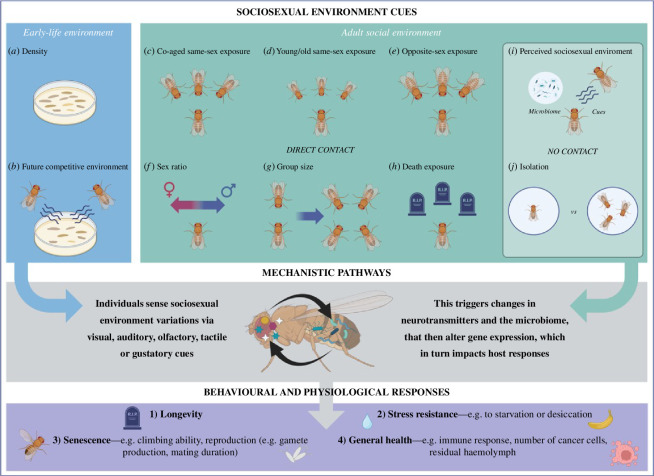
The types of social environment manipulations researchers use to examine social effects on ageing and lifespan using *Drosophila* as an example. Some studies manipulate the early life or developmental social environment, either through density (*a*) or using cues from adults (*b*), and then measure their effects on adult life-history traits. Studies that manipulate the adult social environment tend to manipulate exposure to the same sex (co-aged (*c*), or older/younger (*d*)), the opposite sex (*e*) or both (*f,g*), or even exposure to deceased conspecifics (*h*) to test for sex-specific effects of the social environment on lifespan and ageing. Mechanistic studies tend to manipulate how males and females respond to cues about their social environment (*i*). Finally, many studies tend to use social isolation (*j*) as a ‘control’ social environment to compare the effects of different social manipulations. Individuals detect these cues using multiple senses and respond to them using neuronal, microbial and genetic pathways (grey box). They then plastically adjust their behaviour and physiology to maximize their potential fitness (purple box), with trade-offs occurring between (1) longevity, (2) stress resistance, (3) senescence and (4) general health. Figure created using BioRender.com.

Our systematic review did find some evidence supporting the role of developmental social environment on adult lifespan or ageing (figure 2, electronic supplementary material, table S1). Out of the 80 studies, we identified 6 that manipulated density as a social stress (e.g. [62,68,81,102,114]). These studies generally reported a negative effect of high density on adult lifespan, and this effect was sometimes sex-specific (electronic supplementary material, table S1). For example, Gutiérrez and colleagues [114] tested potential trade-offs under two different stressors: nutritional environment and social environment, in *Acheta domesticus* crickets. They provided crickets with a low or a high diet (unbalanced versus balanced protein:carbohydrate diet) and then manipulated social environment (solitary versus group) throughout adult life stages. An unbalanced diet significantly increased development time for both sexes, increased female fecundity and reduced female lifespan [114]. However, the social environment significantly influenced cricket survival to adulthood in both sexes, whereby group-living crickets had poorer survival outcomes than did solitary crickets [114].

Phenotypic effects of larval density can also translate to behavioural changes in adults that can then affect fitness and longevity. In insects, resources accrued during development directly influence body size, which can then directly determine female fecundity and male competitive ability. For instance, adult *D. melanogaster* raised at low larval densities had higher courtship rates and longer lifespans, but produced offspring with poorer survival outcomes, than flies raised at high larval densities [122]. How insects acquire and invest nutritional resources may change depending on the environment in which they develop and the social environment they experience as adults. Developing individuals can use cues about their future social environment to modulate trait expression. Kasumovic and colleagues [73] manipulated the juvenile social environment (i.e. density) and the perceived future competitive environment by providing juvenile field crickets (*Teleogryllus commodus*) with variable call environments that signalled varying levels of competition (low, high or unpredictable). Call environment influenced age-specific calling effort and the rate of senescence of calling effort in males, and female responses to male calls [73]. Moreover, density, but not call environment, affected male lifespan, with males from lower-density treatments living significantly longer than males from higher-density treatments [73]. Thus, cues received during development can influence the plasticity of trait expression and preferences in adults.

### (c) How do the sexes each respond to same versus opposite sex exposure?

Studies that manipulated the adult social environment (*n* = 61 of the 80 studies) manipulated exposure to the same or opposite sex to test for sex-specific effects of the social environment on lifespan and ageing. More specifically, these studies either manipulated exposure to (i) same-sex rivals (e.g. male–male competitive scenarios; *n* = 21 studies), (ii) the opposite sex (i.e. mating rate or mating costs; *n* = 18 studies) or (iii) varied exposure to both sexes (i.e. sex ratios; *n* = 37 studies). Social exposure generally occurred through physical interactions between individuals, but some studies did manipulate exposure to cues of the same or opposite sex. Interestingly, many studies used social isolation as a ‘control’ social environment. There was no strong sex bias in the focal sex across these 80 studies; 23 studies focused only on male responses to the social environment and 18 focused on females, while the remaining 39 measured the responses of both sexes.

When considering same-sex interactions, male lifespans appear to be more sensitive to same-sex exposure than females. A possible reason for this is that males are generally more aggressive to each other than are females [123,124]. For example, Kudo [112] compared the lifespan of male and female *Drosophila prolongata* flies from four different strains, including a ‘hyperaggressive’ strain, when kept alone or in same-sex groups. Grouping reduced the lifespan of males from the ‘hyperaggressive’ strain to a much greater extent than in other strains. Moreover, the difference in lifespan between these two different social environments for males was greater than that for females, even for the ‘hyperaggressive’ females [112]. In insects, damage accrued during male–male combat is likely to be cumulative as the exoskeleton cannot be easily repaired or replaced. In this way, aggressive male–male interactions can induce somatic deterioration and shorten lifespan. Adler and colleagues [102] experimentally tested the link between male condition, the level of male–male competition and somatic deterioration using neriid flies, *T. angusticollis*. They found that high-condition males (i.e. males optimized for male–male contest ability) that were kept in all male groups suffered higher rates of somatic damage and had shorter lifespans than low-condition males or males kept in mixed-sex groups [102].

Intrinsic male condition can influence success in male–male competition, and hence fitness outcomes. However, males can plastically adjust their resource allocation towards traits associated with reproduction (i.e. fighting ability and mating duration) and traits positively associated with lifespan (i.e. somatic maintenance) in response to their social environment. Indeed, many studies have demonstrated an increased investment into pre- or post-copulatory sexual traits, and an equivalent decrease in male lifespan, in response to same-sex rivals (e.g. [47,66,103]). Lifetime exposure to rival males increased male calling effort in *T. commodus* crickets [98], while *D. melanogaster* males that had been exposed to rivals took longer to mate, but mated for longer, than isolated males [52,94]. These plastic responses to rivals not only occur when there are direct interactions among males, such as male–male contests, but also following exposure to one or more sensory cues of rival presence (e.g. [71,97]). Such plasticity is thought to occur when the sociosexual environment reliably signals either the current or future competitive environment [125]. This suggests that, even in the absence of direct interactions between males, cues of the sociosexual environment can modulate trade-offs between reproduction and lifespan.

While males are more sensitive to the presence of same-sex rivals, females exposed to males can experience dramatic lifespan loss. This may be owing to sex-specific costs of reproduction, whereby the energetic demands of reproduction for females are generally greater than, or different from, those of males [126]. We found that many studies examined these costs of reproduction on male and female lifespan by varying the operational sex ratio (OSR) from heavily female-biased to heavily male-biased (e.g. [57,75,83,89,107]). Females consistently had the longest lifespans when kept alone or with only other females [100,109,117], while increased male exposure decreased female lifespan (e.g. [127,128]). While studies of sex ratios are valuable for quantifying the costs of reproduction for male and female lifespan, we found that far fewer studies then tested how variation in exposure to the opposite sex influenced rates of ageing in both sexes (but see [92,104]). This is an important distinction as the general loss of lifespan associated with increased investment into reproduction might mask more subtle sex differences in mortality risks and the onset of senescence associated with varied exposure to the same and opposite sex. For example, Adler & Bonduriansky [119] found that variation in the OSR had strong, sex-specific effects on lifespan and ageing patterns in *T. angusticollis* flies. For males, early life mortality rate increased, and lifespan decreased, as the OSR became increasingly male-biased. Females, however, consistently lived less long than males regardless of the number of males in their social group [119]. Therefore, studies that examine how males and females each respond to varying social contexts are important for understanding sex differences in ageing and lifespan.

### (d) Age of social partners

Many studies used experimental cohorts with non-focal individuals that are themselves co-ageing with the focal individuals (figure 2, electronic supplementary material, table S1). However, we identified four studies that did use social partners of different ages relative to the focal individual [106,109,110,117]. Social relationships are known to change with age, with older individuals becoming more socially selective by interacting with fewer social partners and being less connected in their social networks (e.g. in forked fungus beetles, *Bolitotherus cornutus* [129]). A further study in *B. cornutus* published in this issue shows that male fitness is reduced by interacting with older males, whereas populations with older age profiles reduce female fitness [130]. It has also been suggested that the age of social partners might influence health outcomes [131]. The studies in *D. melanogaster* that alter social partner age generally find a negative trend between social partners' age and longevity, i.e. young same-sex partners are beneficial but older partners deleterious to lifespan [106,109,110,117]. In addition to extending lifespan, young social partners also seem to increase physiological indicators of health. Both Ruan and Wu [106] and Cho and colleagues [117] showed that exposure to young social ‘friends’ improved the climbing ability and stress resistance in old flies. Likewise, Lin and colleagues [109] found that exposure to old ‘friends’ reduced resistance to environmental stressors, though not to oxidative stress, and decreased male courtship activity. Leech and colleagues [110] (see below) suggest that young social partners have less effect than co-ageing social partners on age-related changes to the microbiome. Additionally, the presence of older non-focal individuals—specifically adult males during larval development—reduced lifespan of both sexes. It is worth noting that these *Drosophila* studies do not isolate the social effects *per se* from consequences or relatedness amongst individuals on lifespan and ageing, so further studies are needed to establish whether such effects are a general phenomenon.

### (e) Mechanisms linking social environments and ageing

There are multiple potential biological pathways linking social environments to ageing [1]. A major advantage of laboratory-based invertebrate studies is that they facilitate mechanistic experimental approaches to understanding how environmental information is mediated through physiological and molecular mechanisms into ageing patterns. Indeed, we found several studies that tackled this through altering the perception of social partners, identifying the involvement of specific genes, genetic pathways and the microbiome (figure 2, electronic supplementary material, table S1).

The effects of an individual's own perceived isolation may differ from those caused by being physically socially isolated. Therefore, the respective sensory inputs operating via potentially different sensory modalities may initiate different responses. In fruit flies, olfaction seems to be a key trait. For example, the cuticular hydrocarbon repertoire of young flies extends lifespan [117] while receipt of female pheromones without the ability to mate reduces male lifespan [97,132]. Moreover, using mutants that are impaired in their olfactory or gustatory senses (the genes *Orco* and *pickpocket*, respectively) reduces the lifespan-extending benefit of young social partners, at least in females [117]. Olfaction also plays a role in the lifespan reduction of females sensing dead conspecifics, but here vision is critical and sufficient to induce lifespan changes [133]. In other traits, sensing and responding to the social environment may use combinations of cues [134]. *D. melanogaster* males require any paired combination of sound, smell and touch in order to mount a response to predicted sperm competition [127], but what is unknown is whether the same sensory inputs also influence lifespan.

Sensing the social environment and initiating behavioural responses to that environment are likely to involve the nervous system. Therefore, neuronal mechanisms could influence ageing patterns. Flintham and colleagues [25] investigated this idea using male *D. melanogaster* flies with feminized nervous systems and female flies with masculinized nervous systems. This had the effect of inducing male-specific courtship behaviour and aggression in masculinized females and male–male courtship and reduced aggression in feminized males. In control flies, consistent with other studies [44,104,113], males suffered reduced lifespan in same-sex groups, whereas females did not. However, females with masculinized nervous systems showed patterns similar to control males. This was likely owing to such individuals receiving male behaviours (e.g. aggression) rather than the cost the cost of producing those behaviours themselves. Flintham *et al*. [25] pointed out that these findings could help to explain why sex differences in ageing trajectories exist. However, other studies have found little evidence that males held with other males suffer aggressive interactions or being excluded from food [32,94]. Activity between social partners seems crucial. Ruan and Wu [106] suggest that the benefits of young helpers are only realized if they have fully functional motor skills and can fully interact with the older partners. Housing with flies carrying mutations in the circadian rhythm gene *period* (defective in daily activity patterns) increases lifespan in females and, notably, males in single sex groups [135]. Therefore, behaviours that are not so intuitively stressful as aggression, such as the amount of activity or the quality of sleep, could be affected when in groups.

A further potential mechanism translating social environments into ageing is the microbiome, and there is increasing attention on the dysbiosis of the microbiome in old age [136]. The ecosystem of microbes that inhabit a host is likely to be altered by the host’s social environment. Social contact aids horizontal transmission of microbes, and social partners are more likely to share similar environments and resources such as food, a major determinant of the microbial community. Indeed, cohabitation, social group membership or social networks can determine microbiome variation in mammals [137]. Stress can feed into the ‘gut–brain axis’, potentially driving microbiome dysbiosis. For example, social stress in mice alters the gut microbial community [138] and isolation behaviours can be induced in socialized mice using faecal transfers from isolated individuals [139]. Such impacts could mediate social effects on ageing, as microbiomes can have substantial effects on host health and ageing patterns [128,140–142]. Leech *et al*. [110] found that same-sex grouping increased bacterial diversity in both sexes. Importantly, the community structure of grouped males became distinctive at older ages. This only occurred if the grouped males were co-aged rather than replenished with young males. This study also identified an effect of the developmental social environment on adult lifespan: being raised with adult males during development reduced subsequent lifespan of both sexes and altered the microbiome at the pupal stage.

While the relationship between socially determined microbiome community structure and lifespan needs further direct testing, Leech *et al*. [110] suggested how these changes to the microbiome could be functionally important. They used known interactions between bacterial genes/metabolites and fly genes to predict the *Drosophila*-specific genetic pathways that would be differentially enriched by changes in microbial community. Focusing on five pathways critical to ageing and immunity, generally sex differences in enrichment were more apparent in groups rather than when alone. Additionally, age had a bigger effect on pathway enrichment when males were grouped with co-ageing rather than young social partners. There is a suggestion that social conditions lead to a conserved transcriptional response: for example, social adversity in humans has a signature of immune- and inflammation-associated gene expression [143]. Certainly, social environments alter gene expression in insects (e.g. [98,144], but the pattern is not always consistent, e.g. [111]). The role of such gene expression changes could be tested with the use of mutants and transgenics (e.g. [106]).

### (f) Linking laboratory and field studies in nature

Almost all of the studies were performed in the laboratory, which could yield contrasting patterns to those found in the field [26]. At present, studies of ageing in the wild are otherwise dominated by those conducted on social mammals (e.g. [2,7–9,38,40]). The longevity of large mammals can be challenging for studies of ageing because many years of continuous observation are required to accrue data. An alternative to long-term studies of wild vertebrate populations is to track the lifespans of invertebrates in the field. A notable example comes from Rodríguez-Muñoz and colleagues [145]. *Gryllus campestris* crickets are one of the few insect systems in which individual adults can be tracked and assessed in the field and this has so far yielded more than 10 years of long-term data [146]. In relation to ageing, Rodríguez-Muñoz and colleagues [145] measured actuarial senescence (change in probability of dying with age) and physical senescence (calling) across years (where there is one discrete generation per year). Across their study period, the sex ratio varied from strongly female-biased to an even sex ratio. Both sexes showed faster actuarial senescence when there were more males in the population. Baseline mortality was higher for females in more female-biased years, consistent with the idea that they are protected from predators when associating with males at their burrows [147]. Males showed a decline in calling with age if there was a more equal sex ratio, in contrast to no senescence in calling when the population was strongly female-biased. This may indicate a cost of competing with rival males. In the closely related cricket *Teleogryllus bimaculatus*, being housed with a rival male reduced lifespan, which the authors attribute to direct interactions between males [98]. Additionally, as mentioned above, in *T. bimaculatus,* hearing other males alters gene expression including in stress/immune response pathways [148] which may have consequences for the lifespan. As sex ratio was not experimentally manipulated in the field, it is difficult to know whether it was causal to the patterns observed; there could have been an environmental variable that altered both sex ratio and ageing. Nevertheless, it is highly suggestive of patterns observed in the laboratory reflecting key components in nature.

Our systematic review has identified a taxonomic bias towards Dipteran species for studies of ageing (figure 1; electronic supplementary material, table S2), which are useful models for laboratory-based, experimental studies. However, there are many other insect species where individuals within a population can potentially be studied over the course of their lifetime [149,150]. Species with high site fidelity can be easily monitored in the field (e.g. *Coenagrion puella* damselflies [151]), while the interactions of group-living invertebrates, like some social spiders, could be studied over time in their communal space (e.g. [152,153]). Moreover, sessile invertebrates, like some marine bryozoans, experience ‘static’ social environments for part of their life (e.g. [154]). Longitudinal studies of such species could provide useful insights into the effects of competition and density on ageing.

## 4. Conclusions and future perspectives

Our systematic review found evidence for social impacts on ageing in ‘non-social’ insects (electronic supplementary material, table S1), which could be tested in the future via meta-analysis. Nevertheless, our review revealed that social environments do have key impacts on lifespan and ageing across a range of invertebrate taxa. First, we expected that negative early life experiences would decrease lifespan. Indeed, several studies that exposed juveniles to high competition did report reduction of lifespan (except for [67,90,96]). Second, we expected to find strong, negative impacts of male presence on female lifespan, but not on male lifespan. There were more studies that focused on the effects of opposite-sex exposure on lifespan than for any other social context, and these studies did tend to report lifespan reduction for females in the presence of males. Interestingly, where social isolation would be assumed to be stressful in studies of humans and other socially complex animals, often this state was described as a control in studies that we identified. It might be fruitful in either group to simply describe differences in social experience, and to assess whether either state is stressful from the outcome of the assays used. Third, studies investigating the effect of same-sex exposure on male lifespan often reported negative impacts of same-sex competition (except when rivals were younger; see [106,109,110,117]), but there was also frequently a negative impact of female competition on female lifespan. These surprising effects warrant further investigation into the costs of same-sex competition in females and whether these are generally associated with social interactions (i.e. dominance interactions) or resource competition.

Social environment impacts were often measured as differences in lifespan but some of the studies that we found measured senescence in physiological traits and behaviours, potentially indicating differences in ‘health span’ [155] (see electronic supplementary material, table S2). Including a range of traits in future studies of senescence would be a major advance because recent studies show that lifespan differences that are dependent on social environment are driven by differences in actuarial senescence (e.g. [156]). A further gap in our knowledge is why sex differences in response to the same environmental manipulations exist [107,156]. The underlying reasons could be addressed by using the mechanistic approaches available for model insects. Indeed, progress has been made in studying social effects mechanistically from sensory inputs to physiological outputs, taking advantage of the relatively easy experimental manipulation and genetic resources available in some species. Future studies could use isolines/strains that differ in their genetic propensity to be social (e.g. [157]) or test for indirect genetic effects (e.g. [107]). Many of the investigations of the effects of social environments have been made in *Drosophila*, and greater taxonomic breadth would be useful. For example, although biomarkers of ageing such as telomeres and DNA methylation are absent in *Drosophila*, these tools are available for other insects [158,159]. Most of the studies reviewed use designs that do not allow individuals to choose their social environment, and it remains unclear whether animals living in fluid groups form stable social networks (as in *Drosophila* [30]). Whilst this may be difficult to examine in natural populations of most insects, semi-natural mesocosm-type experiments could be useful.

## Data Availability

The end point of our systematic review is provided online as a supplementary table, which includes publication information and categorizes aspects of the study designs [160].

## References

[1] Snyder-Mackler N *et al*. 2020 Social determinants of health and survival in humans and other animals. Science **368**, eaax9553. (10.1126/science.aax9553) 32439765 PMC7398600

[2] Albery GF, Clutton-Brock TH, Morris A, Morris S, Pemberton JM, Nussey DH, Firth JA. 2022 Ageing red deer alter their spatial behaviour and become less social. Nat. Ecol. Evol. **6**, 1231–1238. (10.1038/s41559-022-01817-9) 35864228 PMC10859100

[3] Permanyer I, Scholl N. 2019 Global trends in lifespan inequality: 1950-2015. PLoS One **14**, e0215742. (10.1371/journal.pone.0215742) 31048892 PMC6497240

[4] Holt-Lunstad J, Smith TB, Layton JB. 2010 Social relationships and mortality risk: a meta-analytic review. PLoS Med. **7**, e1000316. (10.1371/journal.pmed.1000316) 20668659 PMC2910600

[5] Piolatto M, Bianchi F, Rota M, Marengoni A, Akbaritabar A, Squazzoni F. 2022 The effect of social relationships on cognitive decline in older adults: an updated systematic review and meta-analysis of longitudinal cohort studies. BMC Public Health **22**, 278. (10.1186/s12889-022-12567-5) 35148704 PMC8831686

[6] Patterson SK *et al*. 2024 Early life adversity has sex-dependent effects on survival across the lifespan in rhesus macaques. Phil. Trans. R. Soc. B **379**, 20220456. (10.1098/rstb.2022.0456) 39463249 PMC11513645

[7] Siracusa ER, Pavez-Fox MA, Negron-Del Valle JE, Phillips D, Platt ML, Snyder-Mackler N, Higham JP, Brent LJN, Silk M. 2024 Social ageing can protect against infectious disease in a group-living primate. Phil. Trans. R. Soc. B **379**, 20220462. (10.1098/rstb.2022.0462) 39463240 PMC11528358

[8] Albery GF, Hasik AZ, Morris S, Morris A, Kenyon F, McBean D, Pemberton JM, Nussey DH, Firth JA. 2024 Divergent age-related changes in parasite infection occur independently of behaviour and demography in a wild ungulate. Phil. Trans. R. Soc. B **379**, 20230508. (10.1098/rstb.2023.0508) 39463254 PMC11513643

[9] Campos FA *et al*. 2024 Wild capuchin monkeys as a model system for investigating the social and ecological determinants of ageing. Phil. Trans. R. Soc. B **379**, 20230482. (10.1098/rstb.2023.0482) 39463253 PMC11513648

[10] Brenman‐Suttner DB, Yost RT, Frame AK, Robinson JW, Moehring AJ, Simon AF. 2020 Social behavior and aging: a fly model. Genes Brain Behav. **19**, e12598. (10.1111/gbb.12598) 31286644

[11] Costa JT. 2018 The other insect societies: overview and new directions. Curr. Opin. Insect Sci. **28**, 40–49. (10.1016/j.cois.2018.04.008) 30551766

[12] Salguero-Gómez R. 2024 More social species live longer, have longer generation times and longer reproductive windows. Phil. Trans. R. Soc. B. **379**, 20220459. (10.1098/rstb.2022.0459) 39463247 PMC11513647

[13] Quigley TP, Amdam GV. 2021 Social modulation of ageing: mechanisms, ecology, evolution. Phil. Trans. R. Soc. B **376**, 20190738. (10.1098/rstb.2019.0738) 33678020 PMC7938163

[14] Gordon DM. 2024 The life history of harvester ant colonies. Phil. Trans. R. Soc. B. **379**, 20230332. (10.1098/rstb.2023.0332) 39463251 PMC11528356

[15] Tasaki E, Takata M, Matsuura K. 2021 Why and how do termite kings and queens live so long? Phil. Trans. R. Soc. B **376**, 20190740. (10.1098/rstb.2019.0740) 33678028 PMC7938161

[16] Heinze J, Schrempf A. 2008 Aging and reproduction in social insects a mini-review. Gerontology **54**, 160–167. (10.1159/000122472) 18367827

[17] Collins DH, Prince DC, Donelan JL, Chapman T, Bourke AFG. 2023 Costs of reproduction are present but latent in eusocial bumblebee queens. BMC Biol. **21**, 153. (10.1186/s12915-023-01648-5) 37430246 PMC10334537

[18] Bennett DA, Schneider JA, Tang Y, Arnold SE, Wilson RS. 2006 The effect of social networks on the relation between Alzheimer’s disease pathology and level of cognitive function in old people: a longitudinal cohort study. Lancet Neurol. **5**, 406–412. (10.1016/S1474-4422(06)70417-3) 16632311

[19] Bisschop MI, Kriegsman DMW, van Tilburg TG, Penninx BWJH, van Eijk JTM, Deeg DJH. 2003 The influence of differing social ties on decline in physical functioning among older people with and without chronic diseases: the longitudinal aging study Amsterdam. Aging Clin. Exp. Res. **15**, 164–173. (10.1007/BF03324496) 12889849

[20] Santini ZI *et al*. 2015 Social network typologies and mortality risk among older people in China, India, and Latin America: a 10/66 dementia research group population-based cohort study. Soc. Sci. Med. **147**, 134–143. (10.1016/j.socscimed.2015.10.061) 26575604

[21] Hajek A *et al*. 2022 Social support and health-related quality of life among the oldest old — longitudinal evidence from the multicenter prospective agecode-agequalide study. Qual. Life Res. **31**, 1667–1676. (10.1007/s11136-021-03070-2) 34939147 PMC9098616

[22] DiNapoli EA, Wu B, Scogin F. 2014 Social isolation and cognitive function in Appalachian older adults. Res. Aging **36**, 161–179. (10.1177/0164027512470704) 25650688

[23] Guo S, Wang X, Kang L. 2020 Special significance of non-Drosophila insects in aging. Front. Cell Dev. Biol. **8**. (10.3389/fcell.2020.576571) PMC7536347 33072758

[24] Bailey NW, Moore AJ. 2018 Evolutionary consequences of social isolation. Trends Ecol. Evol. **33**, 595–607. (10.1016/j.tree.2018.05.008) 30055910

[25] Flintham EO, Yoshida T, Smith S, Pavlou HJ, Goodwin SF, Carazo P, Wigby S. 2018 Interactions between the sexual identity of the nervous system and the social environment mediate lifespan in Drosophila melanogaster. Proc. R. Soc. B. **285**, 20181450. (10.1098/rspb.2018.1450) PMC6283938 30487307

[26] Piper MDW, Partridge L. 2018 Drosophila as a model for ageing. Biochim. et Biophys. Acta (BBA) - Mol. Basis Dis. **1864**, 2707–2717. (10.1016/j.bbadis.2017.09.016) 28964875

[27] Tsurumi A, Li WX. 2020 Aging mechanisms—a perspective mostly from Drosophila. Adv. Genet. **1**, e10026. (10.1002/ggn2.10026) 36619249 PMC9744567

[28] Sokolowski MB. 2010 Social interactions in 'simple' model systems. Neuron **65**, 780–794. (10.1016/j.neuron.2010.03.007) 20346755

[29] Chen M, Sokolowski MB. 2022 How Social Experience and Environment Impacts Behavioural Plasticity in Drosophila. Fly **16**, 68–84. (10.1080/19336934.2021.1989248) 34852730 PMC9718549

[30] Wice EW, Saltz JB. 2021 Selection on heritable social network positions is context-dependent in Drosophila melanogaster. Nat. Commun. **12**, 3357. (10.1038/s41467-021-23672-1) 34099680 PMC8185000

[31] Dukas R. 2020 Natural history of social and sexual behavior in fruit flies. Sci. Rep. **10**, 21932. (10.1038/s41598-020-79075-7) 33318613 PMC7736333

[32] Leech T, Sait SM, Bretman A. 2017 Sex-specific effects of social isolation on ageing in Drosophila melanogaster. J. Insect Physiol. **102**, 12–17. (10.1016/j.jinsphys.2017.08.008) 28830760

[33] Dawson EH *et al*. 2018 Social environment mediates cancer progression in Drosophila. Nat. Commun. **9**, 3574. (10.1038/s41467-018-05737-w) 30177703 PMC6120865

[34] Donlea JM, Shaw PJ. 2009 Sleeping together using social interactions to understand the role of sleep in plasticity. Adv. Genet. **68**, 57–81. (10.1016/S0065-2660(09)68003-2) 20109659 PMC2850595

[35] Li W, Wang Z, Syed S, Lyu C, Lincoln S, O’Neil J, Nguyen AD, Feng I, Young MW. 2021 Chronic social isolation signals starvation and reduces sleep in Drosophila. Nature **597**, 239–244. (10.1038/s41586-021-03837-0) 34408325 PMC8429171

[36] Flatt T. 2011 Survival costs of reproduction in Drosophila. Exp. Gerontol. **46**, 369–375. (10.1016/j.exger.2010.10.008) 20970491

[37] Siracusa ER, Negron-Del Valle JE, Phillips D, Platt ML, Higham JP, Snyder-Mackler N, Brent LJN. 2022 Within-individual changes reveal increasing social selectivity with age in rhesus macaques. Proc. Natl Acad. Sci. USA **119**, e2209180119. (10.1073/pnas.2209180119) 36445967 PMC9894112

[38] Woodman J, Gokcekus S, Beck KB, Green JP, Nussey D, Firth JA. 2024 The ecology of ageing in wild societies: linking age structure and social behaviour. Phil. Trans. R. Soc. B **379**, 20220464. (10.1098/rstb.2022.0464) 39463244 PMC11513650

[39] Moiron M, Bouwhuis S. 2024 Age-dependent shaping of the social environment in a long-lived seabird: a quantitative genetic approach. Phil. Trans. R. Soc. B. **379**, 20220465. (10.1098/rstb.2022.0465) 39463241 PMC11513638

[40] Fürtbauer I, Shergold C, Christensen C, Bracken AM, Heistermann M, Papadopoulou M, O’Riain MJ, King AJ. 2024 Linking energy availability, movement and sociality in a wild primate (Papio ursinus). Phil. Trans. R. Soc. B. **379**, 20220466. (10.1098/rstb.2022.0466) 39463242 PMC11513646

[41] Hasenjager M, Fefferman N. 2024 Social ageing and higher-order interactions: social selectiveness can enhance older individuals’ capacity to transmit knowledge. Phil. Trans. R. Soc. B. **379**, 20220461. (10.1098/rstb.2022.0461) 39463239 PMC11513644

[42] Foo YZ, O’Dea RE, Koricheva J, Nakagawa S, Lagisz M. 2021 A practical guide to question formation, systematic searching and study screening for literature reviews in ecology and evolution. Methods Ecol. Evol. **12**, 1705–1720. (10.1111/2041-210X.13654)

[43] Nandy B, Gupta V, Udaykumar N, Samant MA, Sen S, Prasad NG. 2014 Experimental evolution of female traits under different levels of intersexual conflict in Drosophila melanogaster. Evolution **68**, 412–425. (10.1111/evo.12271) 24117169

[44] Nandy B, Gupta V, Sen S, Udaykumar N, Samant MA, Ali SZ, Prasad NG. 2013 Evolution of mate-harm, longevity and behaviour in male fruit flies subjected to different levels of interlocus conflict. BMC Evol. Biol. **13**, 212. (10.1186/1471-2148-13-212) 24073883 PMC3849880

[45] Morimoto J, Ponton F, Tychsen I, Cassar J, Wigby S. 2017 Interactions between the developmental and adult social environments mediate group dynamics and offspring traits in Drosophila melanogaster. Sci. Rep. **7**, 3574. (10.1038/s41598-017-03505-2) 28620201 PMC5472581

[46] Maklakov AA, Bonduriansky R. 2009 Sex differences in survival costs of homosexual and heterosexual interactions: evidence from a fly and a beetle. Anim. Behav. **77**, 1375–1379. (10.1016/j.anbehav.2009.03.005)

[47] Lizé A, Price TAR, Heys C, Lewis Z, Hurst GDD. 2014 Extreme cost of rivalry in a monandrous species: male–male interactions result in failure to acquire mates and reduced longevity. Proc. R. Soc. B **281**, 20140631. (10.1098/rspb.2014.0631) PMC4046415 24827446

[48] Le Page S, Sepil I, Flintham E, Pizzari T, Carazo P, Wigby S. 2017 Male relatedness and familiarity are required to modulate male-induced harm to females in Drosophila. Proc. R. Soc. B **284**, 20170441. (10.1098/rspb.2017.0441) PMC5563793 28794215

[49] Rebar D, Barbosa F, Greenfield MD. 2019 Female reproductive plasticity to the social environment and its impact on male reproductive success. Behav. Ecol. Sociobiol. **73**, 48. (10.1007/s00265-019-2661-4)

[50] Thiéry D, Monceau K, Moreau J. 2014 Larval intraspecific competition for food in the European grapevine moth Lobesia botrana. Bull. Entomol. Res. **104**, 517–524. (10.1017/S0007485314000273) 24788023

[51] Burton-Chellew MN, Sykes EM, Patterson S, Shuker DM, West SA. 2007 The cost of mating and the relationship between body size and fitness in males of the parasitoid wasp Nasonia vitripennis. Evol. Ecol. Res. **9**, 921–934.

[52] Bretman A, Fricke C, Hetherington P, Stone R, Chapman T. 2010 Exposure to rivals and plastic responses to sperm competition in Drosophila melanogaster. Behav. Ecol. **21**, 317–321. (10.1093/beheco/arp189)

[53] Zur T, Nemny-Lavy E, Papadopoulos NT, Nestel D. 2009 Social interactions regulate resource utilization in a tephritidae fruit fly. J. Insect Physiol. **55**, 890–897. (10.1016/j.jinsphys.2009.05.013) 19505473

[54] Xu J, Wang Q. 2014 Ejaculate economics: an experimental test in a moth. Biol. Lett. **10**, 20131031. (10.1098/rsbl.2013.1031) 24429687 PMC3917346

[55] Wilson CJ, Tomkins JL. 2015 Female Callosobruchus maculatus can maximize long-term fitness through polyandry. Behav. Ecol. **26**, 502–509. (10.1093/beheco/aru218)

[56] Wigby S, Chapman T. 2004 Female resistance to male harm evolves in response to manipulation of sexual conflict. Evolution. **58**, 1028–1037. (10.1111/j.0014-3820.2004.tb00436.x) 15212383

[57] White NDG, Bell RJ. 1993 Effects of mating status, sex ratio, and population density on longevity and offspring production of Cryptolestes ferrugineus (Stephens) (Coleoptera: Cucujidae). Exp. Gerontol. **28**, 617–631. (10.1016/0531-5565(93)90051-E) 8137898

[58] VandenBrooks JM, Ford CF, Harrison JF. 2020 Responses to alteration of atmospheric oxygen and social environment suggest trade-offs among growth rate, life span, and stress susceptibility in giant mealworms (Zophobas morio). Physiol. Biochem. Zool. **93**, 358–368. (10.1086/710726) 32758057

[59] Taylor ML, Wedell N, Hosken DJ. 2008 Sexual selection and female fitness in Drosophila simulans. Behav. Ecol. Sociobiol. **62**, 721–728. (10.1007/s00265-007-0497-9)

[60] Sultanova Z, Andic M, Carazo P. 2018 The 'unguarded-X' and the genetic architecture of lifespan: inbreeding results in a potentially maladaptive sex-specific reduction of female lifespan in Drosophila melanogaster. Evolution. **72**, 540–552. (10.1111/evo.13426) 29336481

[61] Saxena S, Mishra G, Omkar. 2020 Operational sex ratio and paternal age sway mating and reproductive performance in Menochilus sexmaculatus (Coleoptera: Coccinellidae). Can. Entomol. **152**, 298–310. (10.4039/tce.2020.14)

[62] Roy SS, Aditya G, Ghosh S. 2018 Impact of density and sex-dependent larval competition on selected life history traits of Drosophila melanogaster (Diptera: Drosophilidae). Can. Entomol. **150**, 87–99. (10.4039/tce.2017.56)

[63] Pascual S, Callejas C. 2004 Intra- and interspecific competition between biotypes B and Q of Bemisia tabaci (Hemiptera: Aleyrodidae) from Spain. Bull. Entomol. Res. **94**, 369–375. (10.1079/ber2003307) 15301702

[64] Parry NJ, Pieterse E, Weldon CW. 2017 Longevity, fertility and fecundity of adult blow flies (Diptera: Calliphoridae) held at varying densities: implications for use in bioconversion of waste. J. Econ. Entomol. **110**, 2388–2396. (10.1093/jee/tox251) 29040631

[65] Paranhos BJ, Ovruski SM, Alves RM, Blummer L, Walder JMM. 2008 Offspring in Response to Parental Female Densities in the Fruit Fly Parasitoid Diachasmimorpha longicaudata (Hymenoptera: Braconidae: Opiinae). Fl. Entomol. **91**, 628–635. (10.1653/0015-4040-91.4.628)

[66] Papadopoulos NT, Liedo P, Müller HG, Wang JL, Molleman F, Carey JR. 2010 Cost of reproduction in male medflies: the primacy of sexual courting in extreme longevity reduction. J. Insect Physiol. **56**, 283–287. (10.1016/j.jinsphys.2009.10.014) 19896949 PMC3018851

[67] Palopoli MF, Peden C, Woo C, Akiha K, Ary M, Cruze L, Anderson JL, Phillips PC. 2015 Natural and experimental evolution of sexual conflict within Caenorhabditis nematodes. BMC Evol. Biol. **15**, 93. (10.1186/s12862-015-0377-2) 25994934 PMC4455605

[68] Onagbola EO, Fadamiro HY, Mbata GN. 2007 Longevity, fecundity, and progeny sex ratio of Pteromalus cerealellae in relation to diet, host provision, and mating. Biol. Control **40**, 222–229. (10.1016/j.biocontrol.2006.10.010)

[69] Okada K, Katsuki M, Kiyose K, Okada Y. 2020 Older males are more competitive in male fights and more aggressive toward females in the broad-horned flour beetle Gnatocerus cornutus. Behav. Ecol. Sociobiol. (Print) **74**, 36. (10.1007/s00265-020-2815-4)

[70] Okada K, Archer CR, Katsuki M, Suzaki Y, Sharma MD, House CM, Hosken DJ. 2015 Polyandry and fitness in female horned flour beetles, Gnatocerus cornutus. Anim. Behav. **106**, 11–16. (10.1016/j.anbehav.2015.05.008)

[71] Noguera JC. 2019 Crickets increase sexual signalling and sperm protection but live shorter in the presence of rivals. J. Evol. Biol. **32**, 49–57. (10.1111/jeb.13390) 30329193

[72] Krueger S, Mound LA, Moritz GB. 2016 Offspring sex ratio and development are determined by copulation activity in Echinothrips americanus MORGAN 1913 (Thysanoptera: Thripidae) . J. Appl. Entomol. **140**, 462–473. (10.1111/jen.12280)

[73] Kasumovic MM, Hall MD, Brooks RC. 2012 The juvenile social environment introduces variation in the choice and expression of sexually selected traits. Ecol. Evol. **2**, 1036–1047. (10.1002/ece3.230) 22837847 PMC3399168

[74] Johansson BG, Jones TM, Widemo F. 2005 Cost of pheromone production in a lekking Drosophila. Anim. Behav. **69**, 851–858. (10.1016/j.anbehav.2004.08.007)

[75] Jehan C, Chogne M, Rigaud T, Moret Y. 2020 Sex-specific patterns of senescence in artificial insect populations varying in sex-ratio to manipulate reproductive effort. BMC Evol. Biol. **20**, 18. (10.1186/s12862-020-1586-x) 32013878 PMC6998128

[76] Janowitz SA, Fischer K. 2010 Costing reproduction: effects of mating opportunity on mating success in male Bicyclus anynana butterflies. Behav. Ecol. Sociobiol. **64**, 1999–2006. (10.1007/s00265-010-1011-3)

[77] Janowitz SA, Fischer K. 2012 Polyandry in Bicyclus anynana butterflies results from sexual conflict over mating. Ethology **118**, 1140–1148. (10.1111/eth.12017)

[78] Izraylevich S, Gerson U. 1995 Sex ratio of Hemisarcoptes coccophagus, a mite parasitic on insects: density-dependent processes. Oikos **74**, 439. (10.2307/3545988)

[79] Iglesias-Carrasco M, Brookes S, Kruuk LEB, Head ML. 2020 The effects of competition on fitness depend on the sex of both competitors. Ecol. Evol. **10**, 9808–9826. (10.1002/ece3.6620) 33005346 PMC7520201

[80] Yu-Bing Huang K, Atlihan R, Gökçe A, Yu-Bing Huang J, Chi H. 2016 Demographic analysis of sex ratio on population growth of Bactrocera dorsalis (Diptera: Tephritidae) with discussion of control efficacy using male annihilation. J. Econ. Entomol. **109**, 2249–2258. (10.1093/jee/tow212) 27694182

[81] Hooper AK, Spagopoulou F, Wylde Z, Maklakov AA, Bonduriansky R. 2017 Ontogenetic timing as a condition-dependent life history trait: High-condition males develop quickly, peak early, and age fast. Evolution **71**, 671–685. (10.1111/evo.13172) 28067402

[82] Hamed M, Sattar M, Nadeem S, Shafique M. 2010 Effect of age on reproduction and sex ratio of Plodia interpunctella (Hubner) (Lepidoptera: Pyralidae). Pak. J. Zool. **42**, 223–226.

[83] Gou Y, Wang G, Quandahor P, Liu Q, Liu C. 2019 Effects of sex ratio on adult fecundity, longevity and egg hatchability of Bradysia difformis Frey at different temperatures. PLoS One **14**, e0217867. (10.1371/journal.pone.0217867) 31166959 PMC6550394

[84] González-López GI, Solís-Echeverría E, Díaz-Fleischer F, Pérez-Staples D. 2019 When less is more: sex ratios for the mass-rearing of Anastrepha ludens (Diptera: Tephritidae). J. Econ. Entomol. **112**, 2997–3001. (10.1093/jee/toz185) 31298285

[85] Gems D, Riddle DL. 2000 Genetic, behavioral and environmental determinants of male longevity in Caenorhabditis elegans. Genetics **154**, 1597–1610. (10.1093/genetics/154.4.1597) 10747056 PMC1461011

[86] Gaskin T, Futerman P, Chapman T. 2002 Increased density and male–male interactions reduce male longevity in the medfly, Ceratitis capitata. Anim. Behav. **63**, 121–129. (10.1006/anbe.2001.1896)

[87] Esfandi K, He XZ, Wang Q. 2015 Flirtation reduces males’ fecundity but not longevity. Evolution. **69**, 2118–2128. (10.1111/evo.12715) 26133013

[88] Dukas R, Yan JL, Scott AM, Sivaratnam S, Baxter CM. 2020 Artificial selection on sexual aggression: correlated traits and possible trade-offs. Evolution **74**, 1112–1123. (10.1111/evo.13993) 32372455

[89] Costa M, Mateus RP, Moura MO, Machado LP de B. 2010 Adult sex ratio effects on male survivorship of Drosophila melanogaster Meigen (Diptera, Drosophilidae). Rev. Bras. entomol. **54**, 446–449. (10.1590/S0085-56262010000300015)

[90] Clutton-Brock T, Langley P. 1997 Persistent courtship reduces male and female longevity in captive tsetse flies Glossina morsitans morsitans Westwood (Diptera: Glossinidae) . Behav. Ecol. **8**, 392–395. (10.1093/beheco/8.4.392)

[91] Charrat B, Amat I, Allainé D, Desouhant E. 2023 Reproductive behaviours in male parasitoids: from mating system to pairing pattern. Ethology **129**, 156–168. (10.1111/eth.13354)

[92] Ceballos S, Kiørboe T. 2011 Senescence and sexual selection in a pelagic copepod. PLoS One **6**, e18870. (10.1371/journal.pone.0018870) 21533149 PMC3077418

[93] Castrezana S, Faircloth BC, Bridges WC, Gowaty PA. 2017 Polyandry enhances offspring viability with survival costs to mothers only when mating exclusively with virgin males in Drosophila melanogaster. Ecol. Evol. **7**, 7515–7526. (10.1002/ece3.3152) 28944035 PMC5606902

[94] Bretman A, Westmancoat JD, Gage MJG, Chapman T. 2013 Costs and benefits of lifetime exposure to mating rivals in male Drosophila melanogaster. Evolution **67**, 2413–2422. (10.1111/evo.12125) 23888861

[95] Brent CS. 2018 Mating and social contact change egg production and longevity in adult females of the mirid Lygus hesperus. Entomol. Exp. Appl. **166**, 545–554. (10.1111/eea.12683)

[96] Filice DCS, Bhargava R, Dukas R. 2020 Plasticity in male mating behavior modulates female life history in fruit flies. Evolution **74**, 365–376. (10.1111/evo.13926) 31925958

[97] Corbel Q, Serra M, García-Roa R, Carazo P. 2022 Male adaptive plasticity can explain the evolution of sexual perception costs. Am. Nat. **200**, E110–E123. (10.1086/720404) 35977789

[98] Callander S, Kahn AT, Hunt J, Backwell PRY, Jennions MD. 2013 The effect of competitors on calling effort and life span in male field crickets. Behav. Ecol. **24**, 1251–1259. (10.1093/beheco/art059)

[99] Berg EC, Lind MI, Monahan S, Bricout S, Maklakov AA. 2019 Kin but less than kind: within-group male relatedness does not increase female fitness in seed beetles. Proc. R. Soc. B **286**, 20191664. (10.1098/rspb.2019.1664) PMC6742989 31506055

[100] Benelli G, Gennari G, Francini A, Canale A. 2013 Longevity costs of same‐sex interactions: first evidence from a parasitic wasp. Invertebr. Biol. **132**, 156–162. (10.1111/ivb.12017)

[101] Athanasiadis K, Pappas ML, Broufas GD. 2021 Effect of duration of exposure to males on female reproductive performance of the green lacewing, Chrysoperla agilis (Neuroptera: Chrysopidae). Insects **12**, 560. (10.3390/insects12060560) 34207007 PMC8234126

[102] Adler MI, Telford M, Bonduriansky R. 2016 Phenotypes optimized for early-life reproduction exhibit faster somatic deterioration with age, revealing a latent cost of high condition. J. Evol. Biol. **29**, 2436–2446. (10.1111/jeb.12968) 27546615

[103] Hughes L, Siew-Woon Chang B, Wagner D, Pierce NE. 2000 Effects of mating history on ejaculate size, fecundity, longevity, and copulation duration in the ant-tended lycaenid butterfly, Jalmenus evagoras. Behav. Ecol. Sociobiol. **47**, 119–128. (10.1007/s002650050002)

[104] Zajitschek F, Zajitschek SRK, Friberg U, Maklakov AA. 2013 Interactive effects of sex, social environment, dietary restriction, and methionine on survival and reproduction in fruit flies. Age **35**, 1193–1204. (10.1007/s11357-012-9445-3) 22798158 PMC3705097

[105] Vogelweith F, Foitzik S, Meunier J. 2017 Age, sex, mating status, but not social isolation interact to shape basal immunity in a group-living insect. J. Insect Physiol. **103**, 64–70. (10.1016/j.jinsphys.2017.10.007) 29038016

[106] Ruan HY, Wu CF. 2008 Social interaction-mediated lifespan extension of Drosophila Cu/Zn superoxide dismutase mutants. Proc. Natl. Acad. Sci. U.S.A. **105**, 7506–7510. (10.1073/pnas.0711127105) 18508973 PMC2396722

[107] Narayan VP, Wilson AJ, Chenoweth SF. 2022 Genetic and social contributions to sex differences in lifespan in Drosophila serrata. J. Evol. Biol. **35**, 657–663. (10.1111/jeb.13992) 35290690 PMC9314142

[108] Morimoto J. 2022 Parental ecological history can differentially modulate parental age effects on offspring physiological traits in Drosophila. Curr. Zool. **68**, 391–399. (10.1093/cz/zoab081) 36090145 PMC9450179

[109] Lin YC, Zhang MY, Wang SH, Chieh CW, Shen PY, Chen YL, Chang YC, Kuo TH. 2022 The deleterious effects of old social partners on Drosophila lifespan and stress resistance. NPJ Aging **8**, 1. (10.1038/s41514-022-00081-2) 35927252 PMC9158773

[110] Leech T, McDowall L, Hopkins KP, Sait SM, Harrison XA, Bretman A. 2021 Social environment drives sex and age-specific variation in Drosophila melanogaster microbiome composition and predicted function. Mol. Ecol. **30**, 5831–5843. (10.1111/mec.16149) 34494339

[111] Leech T, Evison SEF, Armitage SAO, Sait SM, Bretman A. 2019 Interactive effects of social environment, age and sex on immune responses in Drosophila melanogaster. J. Evol. Biol. **32**, 1082–1092. (10.1111/jeb.13509) 31313398

[112] Kudo A. 2021 Intraspecific variation in longevity of Drosophila prolongata (Diptera: Drosophilidae) under solitary and group conditions. Entomol. Sci. **24**, 330–337. (10.1111/ens.12484)

[113] Iliadi KG, Iliadi NN, Boulianne GL. 2009 Regulation of Drosophila life-span: effect of genetic background, sex, mating and social status. Exp. Gerontol. **44**, 546–553. (10.1016/j.exger.2009.05.008) 19481597

[114] Gutiérrez Y, Fresch M, Ott D, Brockmeyer J, Scherber C. 2020 Diet composition and social environment determine food consumption, phenotype and fecundity in an omnivorous insect. R. Soc. Open Sci. **7**, 200100. (10.1098/rsos.200100) 32431901 PMC7211883

[115] Gendron CM, Kuo TH, Harvanek ZM, Chung BY, Yew JY, Dierick HA, Pletcher SD. 2014 Drosophila life span and physiology are modulated by sexual perception and reward. Science **343**, 544–548. (10.1126/science.1243339) 24292624 PMC4042187

[116] Ellen ED, Peeters K, Verhoeven M, Gols R, Harvey JA, Wade MJ, Dicke M, Bijma P. 2016 Direct and indirect genetic effects in life-history traits of flour beetles (Tribolium castaneum). Evolution. **70**, 207–217. (10.1111/evo.12835) 26660947

[117] Cho LC, Yu CC, Kao CF. 2021 Social perception of young adults prolongs the lifespan of aged Drosophila. NPJ Aging Mech. Dis. **7**, 21. (10.1038/s41514-021-00073-8) 34471134 PMC8410773

[118] Carazo P, Green J, Sepil I, Pizzari T, Wigby S. 2016 Inbreeding removes sex differences in lifespan in a population of Drosophila melanogaster. Biol. Lett. **12**, 20160337. (10.1098/rsbl.2016.0337) 27354712 PMC4938057

[119] Adler MI, Bonduriansky R. 2011 The dissimilar costs of love and war: age-specific mortality as a function of the operational sex ratio. J. Evol. Biol. **24**, 1169–1177. (10.1111/j.1420-9101.2011.02250.x) 21375650

[120] Grafen A. 1988 On the uses of data on lifetime reproductive success. In Reproductive success (ed. T Clutton-Brock), pp. 454–471. Chicago, IL: University of Chicago Press.

[121] Cooper EB, Kruuk LEB. 2018 Ageing with a silver-spoon: a meta-analysis of the effect of developmental environment on senescence. Evol. Lett. **2**, 460–471. (10.1002/evl3.79) 30283695 PMC6145406

[122] Morimoto J, Pizzari T, Wigby S. 2016 Developmental environment effects on sexual selection in male and female Drosophila melanogaster. PLoS One **11**, e0154468. (10.1371/journal.pone.0154468) 27167120 PMC4864243

[123] Andersson M. 1994 Sexual selection. Princeton, New Jersey: Princeton University Press.

[124] Wong BBM, Candolin U. 2005 How is female mate choice affected by male competition? Biol. Rev. Camb. Philos. Soc. **80**, 559–571. (10.1017/S1464793105006809) 16221329

[125] Bretman A, Gage MJG, Chapman T. 2011 Quick-change artists: male plastic behavioural responses to rivals. Trends Ecol. Evol. **26**, 467–473. (10.1016/j.tree.2011.05.002) 21680050

[126] Stearns SC. 1989 Trade-offs in life-history evolution. Funct. Ecol. **3**, 259. (10.2307/2389364)

[127] Bretman A, Westmancoat JD, Gage MJG, Chapman T. 2011 Males use multiple, redundant cues to detect mating rivals. Curr. Biol. **21**, 617–622. (10.1016/j.cub.2011.03.008) 21439827

[128] Thomas S *et al*. 2017 The host microbiome regulates and maintains human health: a primer and perspective for non-microbiologists. Cancer Res. **77**, 1783–1812. (10.1158/0008-5472.CAN-16-2929) 28292977 PMC5392374

[129] Cook PA, Costello RA, Formica VA, Brodie ED. 2023 Individual and population age impact social behavior and network structure in a long-lived insect. Am. Nat. **202**, 667–680. (10.1086/726063) 37963123

[130] Cook PA, Costello RA, Brodie ED, Formica V. 2024 Population age structure shapes selection on social behaviour in a long-lived insect. Phil. Trans. R. Soc. B **379**, 20230331. (10.1098/rstb.2023.0331) 39463252 PMC11513641

[131] Muennig P, Jiao B, Singer E. 2018 Living with parents or grandparents increases social capital and survival: 2014 General Social Survey-National Death Index. SSM Popul. Health **4**, 71–75. (10.1016/j.ssmph.2017.11.001) 29349275 PMC5769098

[132] Harvanek ZM, Lyu Y, Gendron CM, Johnson JC, Kondo S, Promislow DEL, Pletcher SD. 2017 Perceptive costs of reproduction drive ageing and physiology in male Drosophila. Nat. Ecol. Evol. **1**, 152. (10.1038/s41559-017-0152) 28812624 PMC5657004

[133] Chakraborty TS, Gendron CM, Lyu Y, Munneke AS, DeMarco MN, Hoisington ZW, Pletcher SD. 2019 Sensory perception of dead conspecifics induces aversive cues and modulates lifespan through serotonin in Drosophila. Nat. Commun. **10**, 2365. (10.1038/s41467-019-10285-y) 31147540 PMC6542802

[134] Dore AA, McDowall L, Rouse J, Bretman A, Gage MJG, Chapman T. 2018 The role of complex cues in social and reproductive plasticity. Behav. Ecol. Sociobiol. **72**, 124. (10.1007/s00265-018-2539-x) 30100665 PMC6060796

[135] Kawaguchi T, Umezaki Y, Ito C, Tomioka K. 2016 Interaction between sexes and between different circadian phenotypes affects lifespan in the fruit fly Drosophila melanogaster. Physiol. Entomol. **41**, 48–58. (10.1111/phen.12124)

[136] Bosco N, Noti M. 2021 The aging gut microbiome and its impact on host immunity. Genes Immun. **22**, 289–303. (10.1038/s41435-021-00126-8) 33875817 PMC8054695

[137] Dowd JB, Renson A. 2018 'Under the skin' and into the gut: social epidemiology of the microbiome. Curr. Epidemiol. Rep. **5**, 432–441. (10.1007/s40471-018-0167-7) 30596004 PMC6290701

[138] Galley JD, Nelson MC, Yu Z, Dowd SE, Walter J, Kumar PS, Lyte M, Bailey MT. 2014 Exposure to a social stressor disrupts the community structure of the colonic mucosa-associated microbiota. BMC Microbiol. **14**, 189. (10.1186/1471-2180-14-189) 25028050 PMC4105248

[139] Gacias M *et al*. 2016 Microbiota-driven transcriptional changes in prefrontal cortex override genetic differences in social behavior. eLife **5**, e13442. (10.7554/eLife.13442) 27097105 PMC4880443

[140] Vaiserman AM, Koliada AK, Marotta F. 2017 Gut microbiota: a player in aging and a target for anti-aging intervention. Ageing Res. Rev. **35**, 36–45. (10.1016/j.arr.2017.01.001) 28109835

[141] Clark RI *et al*. 2015 Distinct shifts in microbiota composition during Drosophila aging impair intestinal function and drive mortality. Cell Rep. **12**, 1656–1667. (10.1016/j.celrep.2015.08.004) 26321641 PMC4565751

[142] Guo L, Karpac J, Tran SL, Jasper H. 2014 PGRP-SC2 promotes gut immune homeostasis to limit commensal dysbiosis and extend lifespan. Cell **156**, 109–122. (10.1016/j.cell.2013.12.018) 24439372 PMC3928474

[143] Cole SW. 2019 The conserved transcriptional response to adversity. Curr. Opin. Behav. Sci. **28**, 31–37. (10.1016/j.cobeha.2019.01.008) 31592179 PMC6779418

[144] Mohorianu I, Bretman A, Smith DT, Fowler EK, Dalmay T, Chapman T. 2017 Genomic responses to the socio-sexual environment in male Drosophila melanogaster exposed to conspecific rivals. RNA **23**, 1048–1059. (10.1261/rna.059246.116) 28428330 PMC5473139

[145] Rodríguez-Muñoz R, Boonekamp JJ, Liu XP, Skicko I, Haugland Pedersen S, Fisher DN, Hopwood P, Tregenza T. 2019 Comparing individual and population measures of senescence across 10 years in a wild insect population. Evolution. **73**, 293–302. (10.1111/evo.13674) 30597539 PMC6590638

[146] Rodríguez-Muñoz R, Bretman A, Slate J, Walling CA, Tregenza T. 2010 Natural and sexual selection in a wild insect population. Science **328**, 1269–1272. (10.1126/science.1188102) 20522773

[147] Rodríguez-Muñoz R, Bretman A, Tregenza T. 2011 Guarding males protect females from predation in a wild insect. Curr. Biol. **21**, 1716–1719. (10.1016/j.cub.2011.08.053) 21982592

[148] Pascoal S, Liu X, Fang Y, Paterson S, Ritchie MG, Rockliffe N, Zuk M, Bailey NW. 2018 Increased socially mediated plasticity in gene expression accompanies rapid adaptive evolution. Ecol. Lett. **21**, 546–556. (10.1111/ele.12920) 29441668

[149] Bonduriansky R, Brooks RJ. 1999 Why do male antler flies (Protopiophila litigata) fight? The role of male combat in the structure of mating aggregations on moose antlers. Ethol. Ecol. Evol. **11**, 287–301. (10.1080/08927014.1999.9522829)

[150] Zajitschek F, Zajitschek S, Bonduriansky R. 2020 Senescence in wild insects: key questions and challenges. Funct. Ecol. **34**, 26–37. (10.1111/1365-2435.13399)

[151] Sherratt TN, Laird RA, Hassall C, Lowe CD, Harvey IF, Watts PC, Cordero-Rivera A, Thompson DJ. 2010 Empirical evidence of senescence in adult damselflies (Odonata: Zygoptera). J. Anim. Ecol. **79**, 1034–1044. (10.1111/j.1365-2656.2010.01719.x) 20584095

[152] Parthasarathy B, Joshi CH, Kalyadan SS, Somanathan H. 2019 Early ontogenic emergence of personality and its long-term persistence in a social spider. Behav. Ecol. Sociobiol. **73**, 35. (10.1007/s00265-019-2645-4)

[153] Beleyur T, Bellur DU, Somanathan H. 2015 Long-term behavioural consistency in prey capture but not in web maintenance in a social spider. Behav. Ecol. Sociobiol. **69**, 1019–1028. (10.1007/s00265-015-1915-z)

[154] Thompson M, Marshall D, Monro K. 2015 Non-contact competition in a sessile marine invertebrate: causes and consequences. Mar. Ecol. Prog. Ser. **522**, 115–125. (10.3354/meps11178)

[155] Partridge L, Deelen J, Slagboom PE. 2018 Facing up to the global challenges of ageing. Nature **561**, 45–56. (10.1038/s41586-018-0457-8) 30185958

[156] Rostant WG, Mason JS, West N, Maklakov AA, Chapman T. 2023 Sociosexual exposure has opposing effects on male and female actuarial senescence in the fruit fly Drosophila melanogaster. J. Gerontol. A Biol. Sci. Med. Sci. **78**, 2230–2239. (10.1093/gerona/glad215) 37694551 PMC10692434

[157] Wice EW, Saltz JB. 2023 Indirect genetic effects for social network structure in Drosophila melanogaster. Philos. Trans. R. Soc. B **378**, 20220075. (10.1098/rstb.2022.0075) PMC9939268 36802774

[158] Kuznetsova V, Grozeva S, Gokhman V. 2019 Telomere structure in insects: a review. J. Zool. Syst. Evol. Res. **58**, 127–158. (10.1111/jzs.12332)

[159] Mason JM, Frydrychova RC, Biessmann H. 2008 Drosophila telomeres: an exception providing new insights. Bioessays **30**, 25–37. (10.1002/bies.20688) 18081009 PMC2804870

[160] Harrison LM, Churchill ER, Fairweather M, Smithson CH, Chapman T, Bretman A. 2024 Data from: Ageing effects of social environments in ‘non-social’ insects. Figshare. (10.6084/m9.figshare.c.7452086) PMC11513649 39463243

